# The effects of fatigue on linear and angular kinematics during bilateral squat

**DOI:** 10.1371/journal.pone.0289089

**Published:** 2023-11-27

**Authors:** Berkant Erman, Faik Vural, Milivoj Dopsaj, Mehmet Zeki Ozkol, Damla Ercan Kose, Tolga Aksit

**Affiliations:** 1 Department of Coaching Education, Institute of Health Sciences, Ege University, Izmir, Turkey; 2 Coaching Education Department, Faculty of Sport Sciences, Ege University, Izmir, Turkey; 3 Faculty of Sport and Physical Education, University of Belgrade, Belgrade, Serbia; 4 Institute of Sport, Tourism and Service South Ural State University, Chelyabinsk, Russia; 5 Physical Education and Sport, Istanbul Technical University, Istanbul, Turkey; Università degli Studi di Milano: Universita degli Studi di Milano, ITALY

## Abstract

This study was aimed to analyze in detail how the fatigue effects to kinematic parameters of body weight squat exercise (BSQ) by dividing a squat cycle into four different regions. Twenty-one male athletes participated in this study. Participants were divided into two groups according to their lower limb muscle ratio (LLMR). The BSQ was performed until participants were unable to continue the exercise due to the fatigue. Linear and angular kinematics were obtained by motion analysis software which has high validity and reliability. There was no significant but had large effect size interaction between fatigue conditions and LLMR groups in terms of knee ROM in the extension phase and hip angular velocity in braking phase of the flexion (0.08 > p >0.05, 0.18 > $\eta_{\rho }^{2}$ > 0.16). Fatigue condition did not have a significant effect on the duration in the acceleration and braking phases of BSQ (p > 0.05). There were many significant main effects on kinematics in the different regions due to the fatigue (0.01 < p <0.05, 0.44 > $\eta_{\rho }^{2}$ > 0.14). In the fatigue condition, there was a polynomial relationship between velocity of shoulder and hip joints (R^2^_flex_ = 0.82, R^2^_ext_ = 0.72) rather than linear (R^2^_flex_ = 0.64, R^2^_ext_ = 0.53) and coefficient correlations also decreased (*r*_flex_ = 0.88 to 0.80, *r*_ext_ = 0.92 to 0.73). The sticking region was observed in the non-fatigue condition and disappeared when fatigue occurred. These results suggest that LLMR may be taken into consideration in the squat exercises, joint tracking may vary for velocity-based squat training and pre-test for sticking region observation may be apply with the BSQ.

## Introduction

The bilateral squat is one of the most common exercises of developing strength [1, 2]. It also plays a vital role in the completion of everyday activities. Therefore, it is one of the most preferred exercises especially for the general population, beginners and athletes to rehabilitation and athletic development [3]. It can be a form of rehabilitation as well [4]. As squat is dynamic and closed chain, it is also influenced by a multitude of factors. The depth of the movement, positions of the feet on the ground, the strength of core muscles, the position of the head may alter the activation of muscles [2, 5–7]. These are important factors that vary according to the individuals’ goals. Additionally, men and women exhibit different motor strategies across the kinematic chain [8].

Fatigue is another critical factor in squat exercises [5, 9, 10]. It has been shown in many studies that there are changes in the kinematics and kinetics of the movement due to the fatigue. Some of these; more forward leaning [6], forward shifting of the ground reaction force [11], reduction of range of motions [3] and decelerate the linear velocity of the joints [12]. In resistance exercises, a phenomenon occurs in the fatigue condition. This phenomenon is called the “stick region”. This region may be simplest summarized as the short-term slowdown and followed by acceleration during the lift (in the ascent phase). The method used in the studies is generally focused on the concentric phase related to this region. Because, the unsuccessful end of the movement or the observation of this “region” occurs at this stage [13]. Even though much have been investigated so far, it is still not possible to say the exact reasons why this phenomenon occurred. In addition to the these, it is not possible to observe this region for each participant in the fatigue condition [13, 14]. Also, in this region, the acceleration and braking durations in the ascent phases are generally not equal and it is observed that the deceleration duration increases more with light external loads [15, 16]. Previous studies haven’t emphasized the body weight squat exercise (BSQ) in exploring these phenomena. However, based on existing findings, it could be expected that the acceleration and braking durations would become more similar in this exercise.

Another important training effect related to squat is velocity-based training. Regarding the velocity of the joints, monitoring the bar speed and predicting one-repetition maximum (1-RM) methods have become very important and popular for the velocity-based training. However, BSQ was not investigated as much as weighted squats (i.e., back squat). Therefore, questions such as how the velocity change of the joints (i.e., hip, shoulder) during the exercise and whether the relationship between them is linearity or not, has not been fully answered. Another situation that needs to be considered here is that monitoring to the bar speed for upper extremity exercises can be more direct method (i.e., bench press). However, while the difference of joint velocities related to this situation has been proven [17, 18], following the lower extremity development or training from monitoring the bar speed may be evaluated indirect method for a multi-joint movement (i.e., squat). In addition, lower limb muscle ratio (LLMR) has an important role in activities such as squatting, jumping, sprinting etc. Although the issue of how the LLMR develops with the different type squat exercises have been well researched, studies of how the LLMR affects the kinematics while the squatting are very limited.

Given these gaps, investigating i) joint velocity kinematics during BSQ, ii) kinematic changes in participants with different LLMRs, and iii) comparing these kinematics in non-fatigue and fatigue conditions, can provide insights. The null hypothesis of this study, there was no tend to significant difference between the LLMR groups in terms of linear and angular kinematics. Therefore, the purpose of this study was to analyze in detail how the fatigue effects to kinematic parameters of BSQ by dividing a squat cycle into four different regions.

## Materials and methods

### Participants

There were more men than women who met the inclusion criteria of this study. Therefore, all participants were selected as men to maintain group homogeneity. Twenty-one recreationally male athletes (age: 23.7±3.2 years (mean ± SD), height: 179.6±7.7 cm, body mass: 79.52±11.78 kg) volunteered and gave their informed consent to take part in the study. All participants with a history of resistance exercise for at least two years (2 or 3 times per week). The inclusion criteria for this study were defined as *(a)* had already practiced and continued squat exercises in a resistance training program, *(b)* had no chronic limb discomfort or limitations that had a negative effect on BSQ kinematics (e.g., pes planus). The exclusion criteria defined as experiencing any injury during the BSQ and not being able to perform BSQ until exhausted. Participants in the study were subdivided into two groups according to the lean muscle mass of the lower limb. These data obtained from a body composition analysis device (Tanita BC 418, Tanita Corp., Tokyo, Japan) which is using the bioelectric impedance method in order to reveal the effect of lower limb muscle mass ratio in the fatigue condition. Based on this, the lean muscle mass (lower limb) data were divided into two groups as low and high by making cut off at 50%. The range of the low group (n_low_ = 10) was 32.00%-33.86% and mean = 33.28% and the range of high group (n_high_ = 11) was 34.25%-36.63% and mean = 34.85%. The range of the whole group (n = 21) was 4.62%. The experimental procedures undertaken were approved by Ege University’s Medical Research Ethics Committee and are in agreement with the principles of the Declaration of Helsinki. The participants were briefed on the study procedures and informed consent was obtained before they took part in the study.

### Instrumentation and data processing

**Fig 1:**
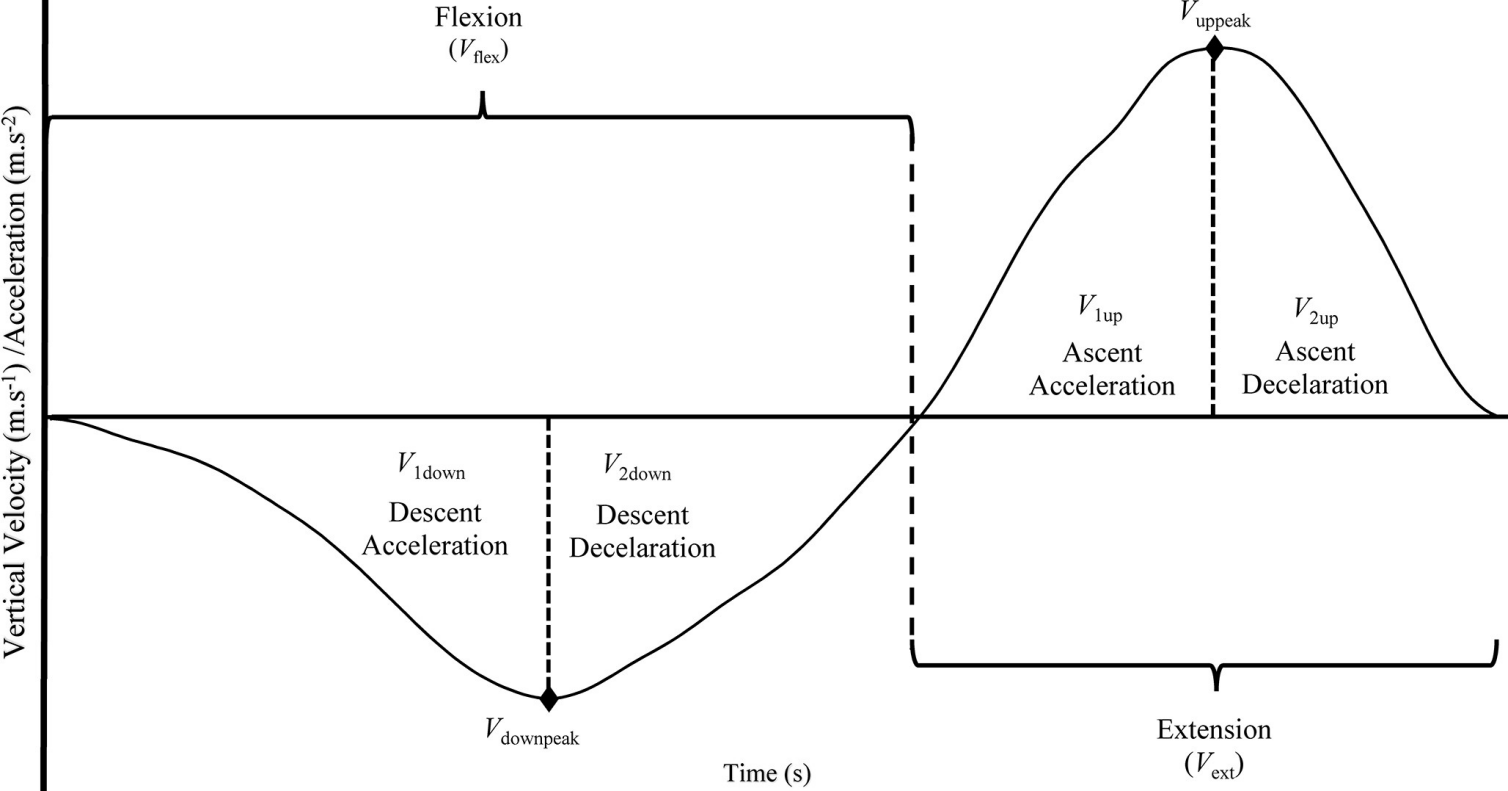
Regions for body weight squat exercise.

The BSQ was performed using a tempo standardized to a two-count cadence at 45 beats per minute (bpm). The cadence was 45 bpm (1:0:1:0) which signifies a one-count eccentric motion followed immediately by a one-count concentric motion, without any count for rest before beginning the next repetition. Linear and angular kinematics of the shoulder, hip and knee joints were examined during the performance. Escamilla et al., (2001) previously described as i) descent phase, ii) acceleration phase, iii) sticking region, iv) maximum strength region and v) deceleration phase for a squat cycle with external load. However, since this study is performed with only body weight, a squat cycle divided into four different regions. These regions were generated in order to make a body weight squat-specific assessment (described below, Fig 1). Another important reason is that the sticking region is not expected to be observed in BSQ as mentioned above. To assessment for kinematic effects of BSQ; the third repetition after the initiation and the previous one before the last accomplished repetition were analyzed in order to observe the movement pattern properly (total; two). The ROMs (°), angular velocities (rad.s^-1^), vertical velocities (m.s^-1^), vertical accelerations (m.s^-2^) and time (s) analyzed for each region. Vertical linear and angular velocities (m.s^-1^, rad.s^-1^), ROMs (°) and accelerations (m.s^-2^) except peak velocities, angles and time were averaged. The first position of upward position (neutral standing posture, start-position) took as initial angle via motion analysis software (full extension, initial angle for hip and knee:180°, ankle:90°), the last point of downward position (descent) evaluated as flexion phase and from the last point of downward position to the last point of upward position evaluated as extension phase. The differences between these angles allowed us to obtain the ROM of hip, knee [180° minus the last point of downward angle (*V*_flex_ region, ROM_flex_); the last point of downward position angle minus 180° (*V*_ext_ region, ROM_ext_); angles between; acromion- trochanter major for hip, trochanter major- malleolus lateralis for knee, epicondylus lateralis- (malleolus lateralis)- malleolus lateralis for ankle]. This study was carried out in a laboratory in two sessions at 72-hour intervals. In the first session, after the participants had done a 10-minute warm up, retroreflective markers were positioned for the acromion (shoulder), trochanter major (hip), epicondylus lateralis (knee), malleolus lateralis (ankle) and caput metatarsalis (toe) points before the exercise. The BSQ was performed for adapt to the metronome and markers. Those who could not adapt to the metronome were excluded from the study. In the second laboratory session, retroreflective markers were placed at the same reference points as in the first laboratory session. The BSQ was captured by a Basler Ace acA1300-200uc camera (Basler AG, Ahrensburg, Germany) with a frequency of 100 Hz (0.01 s). A digital metronome was used to standardize the participants’ BSQ pace (45 bpm, 1:0:1:0). The camera was placed on the right lateral side of the participants (dominant leg), distance: ~2m, camera angle according to the participant: 90° [19]. After the participants had enough warmed up, they performed the BSQ looking straight ahead at a fixed point that had been placed on the wall with their feet approximately opened hip width (greater trochanter width) with feet externally rotated to a maximum of 15°. Because, when comparing 2-D and 3-D analysis for the squat exercises, foot angle (i.e., 0–15°) and narrow stance (107±10% of shoulder width) form of squat are showed minimum error between 2-D and 3-D analysis (Escamilla et al., 2001). For this reason, in this study, squat performed that can be called “narrow stance” was carried out. The participants raised their arms at the descent phase and released their arms the arms during the ascent phase to avoid the fatigue of upper limb. The flexion phase of the squat was not limited. We identified fatigue as a situation in which participan’s joint kinematics are severely disrupted, failed to keep up with the metronome and cannot continue the exercise (unable to squat or stand up).

In the various studies, the Kinovea motion analyses software^®^ was used for kinematic analysis such as vertical jumps [20], running (kappa = 0.76–0.92) [21], ballet dancers (ICC = 0.96–0.99) [22]. A study compared with VICON Motion System^®^ (hip, knee, ankle joints, ICC > 0.85; ICC for the IRR was > 0.90) [23] for gait analysis. Therefore, kinematics of 2-D data were analysed with Kinovea motion analyses software^®^ (v0.9.4, downloaded at: www.kinovea.org, accessed on 03 November 2020) for the present study.

### Defining the regions for BSQ

In order to examine each phase of the BSQ more details, a repetition is divided into four regions (Fig 1). The first region was from the beginning of flexion (full extension) to the peak velocity of flexion (*V*_1down_). Acceleration values of first region was also called “Descent Acceleration”. The second region (*V*_2down_) was from peak velocity of flexion (*V*_downpeak_) to the end of flexion. Acceleration of second region corresponding to the same phase denotes "Descent Deceleration ". The third region (*V*_1up_) started from beginning of extension to peak extension velocity (*V*_uppeak_), acceleration values of third region was also called “Ascent Acceleration”. The last region was from peak velocity of extension (*V*_uppeak_) to end of extension which was also called *V*_2up_ (Fig 1). The averaged acceleration value corresponding to the same phase denotes "Ascent Deceleration". All velocity (m.s^-1^, rad.s^-1^) and accelerations (m.s^-2^) except peak velocities, angles and time were averaged.

### Statistical analysis

The present investigation was designed as a cross-sectional, between- within subject study. The sample size was calculated using GPower 3.1.9.7 [power size (1-β) = 0.93, the effect size (f) = 0.40, type-1 error (α) = 0.05, number of groups = 2, number of measurements = 2, cor. among rep. measure = 0.5, correction ε = 1, input/direct parameter $\eta_{\rho }^{2}$ = 0.14]. All negative variables are shown as positive by taking absolute values. To assess differences between the 2 squat conditions (non-fatigue, fatigue) x 2 LLMR group (low-high), analyses of variance (two-way mixed ANOVA) was performed. Greenhouse-Geisser adjustments of the *p*-values were reported due to sphericity assumption was violated [24]. Partial eta squared ($\eta_{\rho }^{2}$) was used as an effect size and categorised as small (0.01–0.06), medium (0.06–0.14) and large effect (> 0.14) [25]. Test-retest reliability for the linear and angular kinematics between the non-fatigue and fatigue conditions was analysed by ICC. The ICC are reported with 95% of confidence interval (95%CI) and categorized as poor (< 0.5), moderate (0.5–0.75), good (0.75–0.9) and excellent (> 0.9) [26]. The linear and polynomial regressions were performed to establish the velocity relationship between shoulder and hip joints in fatigue conditions. Pearson correlation coefficients (r) were also used to determine the significance of the velocity of shoulder and hip joints relationships. Statistical analysis and graphics were performed by using RStudio 1.3 [27] with five packages [28–32]. The regression graphics created with Microsoft Excel (v16.0, Microsoft Corporation, WA, USA). The statistical significance level was set at *p* ≤ .05.

## Results

**Fig 2:**
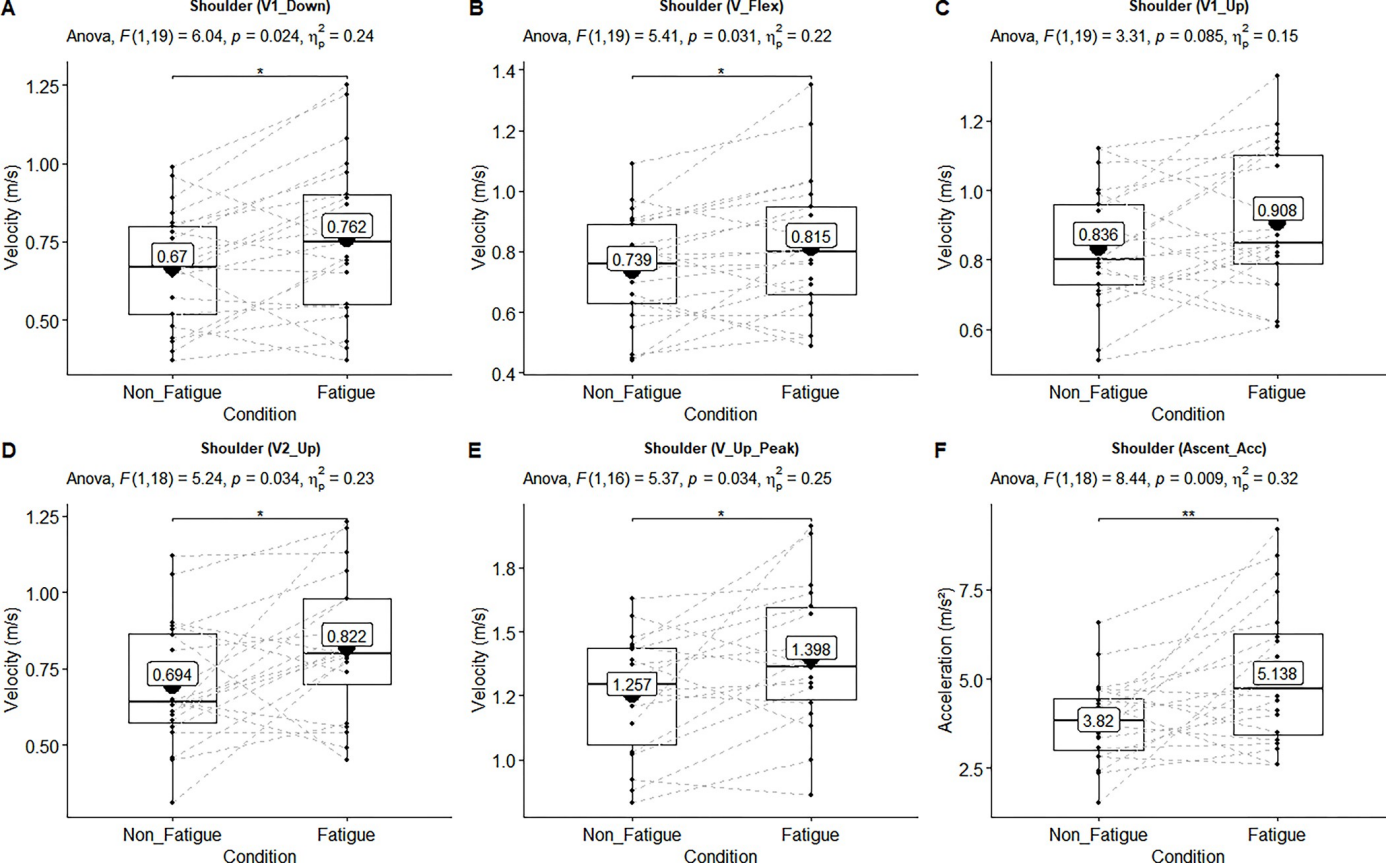
Main effect of the linear velocities of the shoulder joint. ANOVA results showed upon each figure. Black lines of box plots in these figures represent medians. The numbers inside the boxplots represent the mean velocities. There was significant main effect of velocities in the “A-B-D-E-F” (p ≤ .05, $\eta_{\rho }^{2}$ > .14). There was not significant but had large effect size in the “C” (p > .05, $\eta_{\rho }^{2}$ > .14).

**Fig 3:**
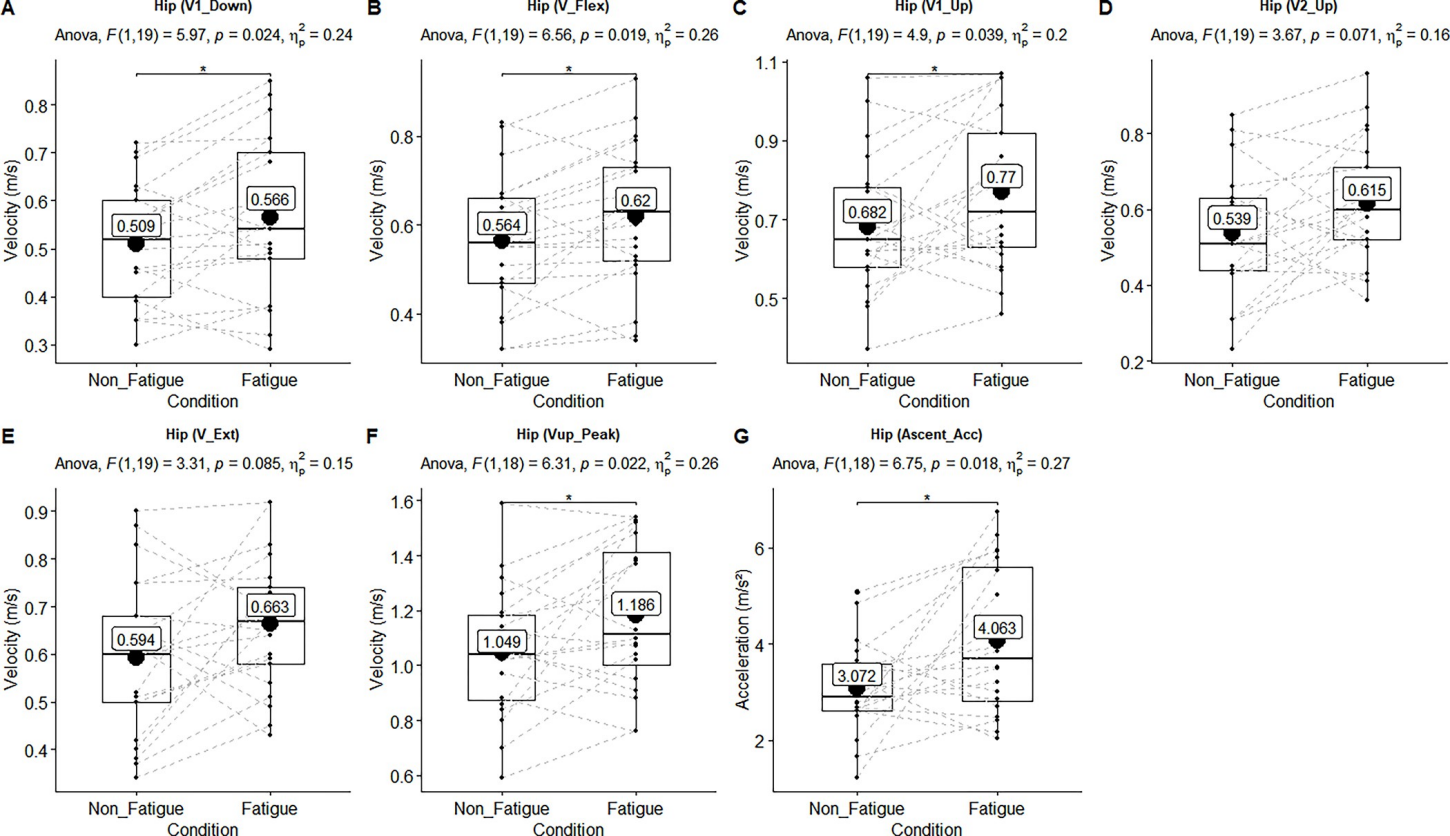
Main effect of the linear velocities of the hip joint. ANOVA results showed upon each figure. Black lines of box plots in these figures represent medians. The numbers inside the boxplots represent the mean velocities. There was statistically significant main effect of the velocities in the “A-B-C-F-G” (p ≤ .05, $\eta_{\rho }^{2}$ > .14). There was not significant but had large effect size in the “D-E” (p > .05, $\eta_{\rho }^{2}$ > .14).

**Fig 4:**
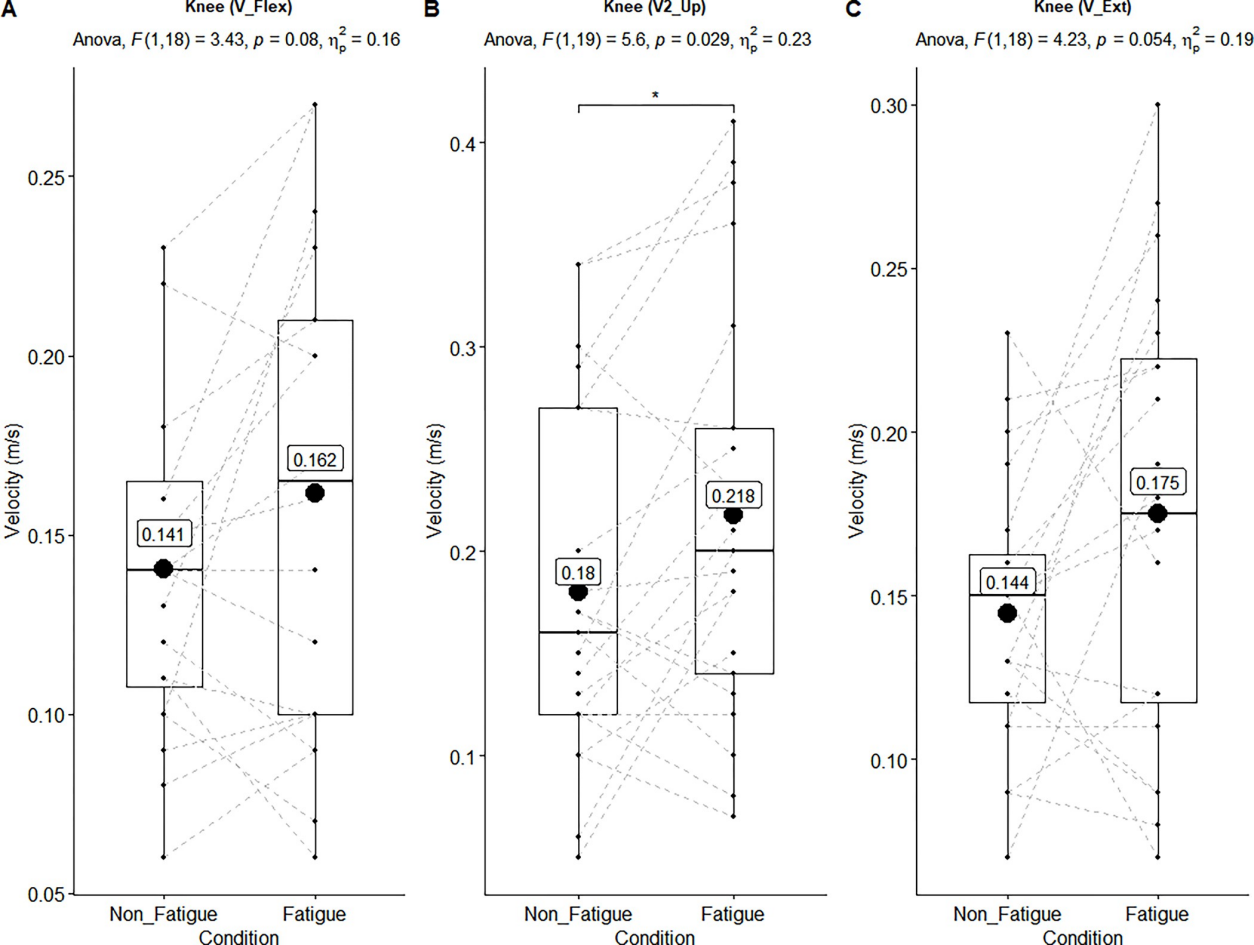
Main effect of the linear velocities of the knee joint. ANOVA results showed upon each figure. Black lines of box plots in these figures represent medians. The numbers inside the boxplots represent the mean velocities. There was statistically significant main effect of the velocities in the “A-B” (p ≤ .05,$\eta_{\rho }^{2}$ > .14). There was not significant but had large effect size in the “C” (p > .05, $\eta_{\rho }^{2}$ > .14).

**Fig 5:**
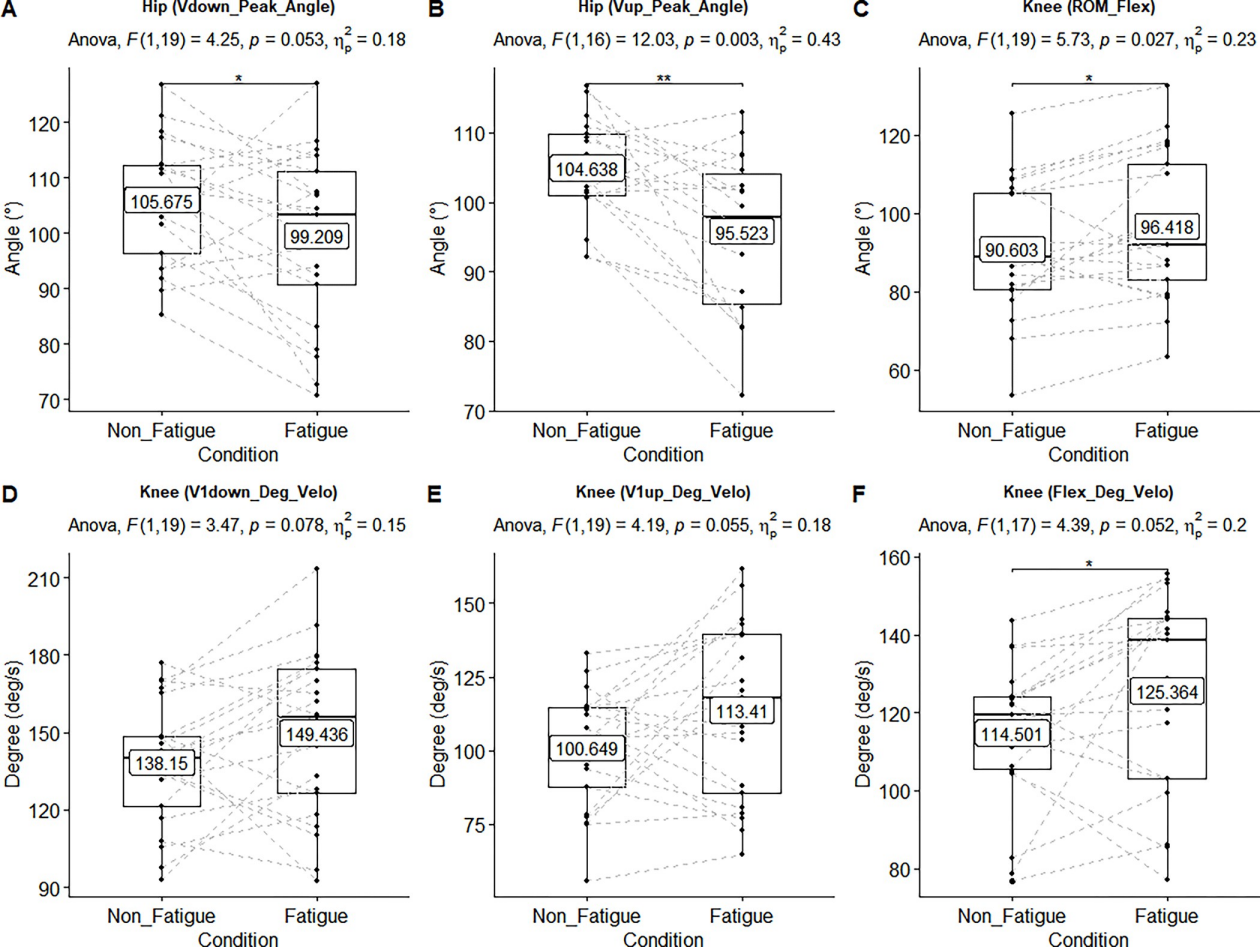
Main effect of the angular velocities of the hip and knee joints. ANOVA results showed upon each figure. Black lines of box plots in these figures represent medians. In the “A-B” represent peak angles were observed. In the “C”, it represents the ROM of knee joint. In the “D-E-F”, they represent mean velocity of knee joint in the regions. There was statistically significant main effect of the velocities in the “B-C” (p ≤ .05, $\eta_{\rho }^{2}$ > .14). There was not significant but had large effect size in the “A-D-E-F” (p > .05, $\eta_{\rho }^{2}$ > .14).

In this study’s analysis; data were screened for assumptions and outliers. Out of twenty-six significant data, nine were outlier as assessed by boxplot. When the results were compared with the previous results (with-outlier), only small differences were observed. Nevertheless, corrected data (without-outlier) were reported. There were also eight non-significant (.08 > p > .05) but large effect size variable ($\eta_{\rho }^{2}$ > .14). These variables are also included in the report (Figs 2C, 3D–3E, 4C and 5D–5F). There was homogeneity of variances (p > .05) and covariances (p > .05), as assessed by Levene’s test of homogeneity of variances and Box’s M test, respectively. Participants performed 72 ± 27 repetitions during the whole exercise. However, only two repetitions were taken into account as described above (non-fatigue vs fatigue).

The results of the two-way mixed ANOVA showed that there was no significant (p > .05) but had large effect size ($\eta_{\rho }^{2}$ > .14) interaction between fatigue condition (non-fatigue vs fatigue) and LLMR groups (low vs high) in terms of knee ROM extension F_(1,19)_ = 4.18, p = .056, $\eta_{\rho }^{2}$ = .17, CI_%90_[0.001–0.401]. In addition, there was also no significant but had large effect size interaction between fatigue condition and LLMR groups in terms of hip degree velocity at *V*_2down_ region F_(1,19)_ = 3.72, p = .069, $\eta_{\rho }^{2}$ = .16, CI_%90_[0.01–0.385]. There was not another significant interaction between fatigue conditions (non-fatigue vs fatigue) and LLMR groups (low vs high) in this study (Table 1).

**Table 1:** Group differences in the same condition and interactions of two-way mixed ANOVA.

	Region		*Joint*											
			Shoulder				Hip				Knee			
			NF		F		NF		F		NF		F	
			H	L	H	L	H	L	H	L	H	L	H	L
Linear	*V*_1down_	m.s^-1^	0.62±0.17	0.72±0.19	0.71±0.25	0.81±0.26	0.48±0.11	0.54±0.13	0.54±0.19	0.58±0.15	0.18±0.09	0.18±0.06	0.20±0.01	0.21±0.09
	*V*_downpeak_		1.14±0.23	1.36±0.40	1.20±0.28	1.36±0.41	0.90±0.20	1.05±0.30	0.95±0.22	1.05±0.27	0.24±0.12	0.24±0.11	0.27±0.14	0.27±0.14
	*V*_2down_		0.76±0.17	0.84±0.20	0.80±0.20	0.90±0.26	0.59±0.16	0.65±0.18	0.65±0.20	0.69±0.17	0.15±0.14	0.11±0.05	0.12±0.07	0.12±0.05
	*V*_flex_		0.70±0.17	0.78±0.19	0.75±0.20	0.88±0.24	0.53±0.14	0.60±0.15	0.59±0.17	0.65±0.14	0.14±0.06	0.15±0.05	0.17±0.08	0.17±0.06
	*V*_1up_		0.80±0.15	0.87±0.19	0.89±0.17	0.92±0.24	0.67±0.16	0.69±0.19	0.77±0.18	0.76±0.22	0.13±0.06	0.12±0.04	0.16±0.09	0.13±0.06
	*V*_uppeak_		1.27±0.31	1.40±0.31	1.42±0.32	1.56±0.40	1.06±0.28	1.11±0.30	1.17±0.26	1.23±0.29	0.24±0.11	0.27±0.10	0.16±0.09	0.13±0.06
	*V*_2up_		0.69±0.18	0.71±0.24	0.82±0.19	0.90±0.34	0.53±0.15	0.54±0.20	0.61±0.13	0.62±0.20	0.17±0.07	0.19±0.10	0.22±0.09	0.21±0.11
	*V*_ext_		0.73±0.17	0.76±0.19	0.85±0.18	0.87±0.26	0.59±0.15	0.60±0.20	0.67±0.14	0.65±0.14	0.15±0.06	0.15±0.04	0.19±0.08	0.17±0.07
	Descent_Acc	m.s^-2^	2.93±1.42	3.40±1.81*	2.95±1.30	3.40±2.37*	2.36±1.04	2.77±1.15*	2.44±1.00	3.09±1.46*	0.61±0.43	0.63±0.35	0.78±0.44	0.82±0.45
	Descent_Dec		2.97±0.84	4.15±1.69	3.28±1.44	4.12±1.91	2.32±0.80	3.20±1.32	2.55±0.81	3.29±1.58	0.60±0.28	0.65±0.40	0.79±0.37	0.79±0.35
	Ascent_Acc		3.97±1.94	4.17±1.25	5.04±1.90	5.32±2.08	3.34±1.77	3.28±1.08	4.20±1.55	3.98±1.50	1.07±0.97	0.85±0.32	1.05±0.54	0.93±0.42
	Ascent_Dec		2.98±0.75	3.35±1.61	3.77±2.14	3.86±2.20	2.52±0.69	2.84±0.98	3.18±1.70	3.00±1.34	0.57±0.26	0.67±0.35	0.82±0.57	0.71±0.53
Angular	*V*_downpeak__Angle	(°)	Not measured.				101.34±10.27	110.43±10.34*	93.09±17.48	107.02±8.98*	105.78±17.61	113.51±11.72	101.78±21.02	113.4±15.04
	*V*_uppeak__Angle						98.19±9.930	109.70±6.96*	88.86±11.19	100.74±8.44*	104.41±18.30	115.65±11.36	99.25±18.67	108.93±11.91
	ROM_Flex						102.03±9.79	99.72±16.24	105.27±17.07	102.10±11.46	89.38±18.19	91.93±16.24	92.78±18.42	100.41±18.84
	ROM_Ext						101.93±10.19	98.85±16.06	100.83±14.86	101.85±12.64	**91.38±19.43**	**90.48±12.81**	**90.11±18.30**	**100.65±16.10**
	*V*_1down__Deg_Velo	rad.s^-1^ (ω)					2.83±0.6	2.57±0.72	2.89±0.73	2.91±1.02	2.43±0.42	2.38±0.45	2.49±0.54	2.73±0.58
	*V*_2down__Deg_Velo						**1.85±0.38**	**1.69±0.47**	**1.44±0.74**	**1.87±0.51**	1.61±0.44	1.5±0.36	1.61±0.47	1.81±0.42
	Flex_Deg_Velo						2.32±0.43	2.16±0.54	2.36±0.57	2.35±0.59	2.01±0.43	1.96±0.35	2.07±0.51	2.24±0.42
	*V*_1up__Deg_Velo						1.89±0.33	1.88±0.46	1.87±0.49	2.0±0.4	1.78±0.39	1.75±0.33	1.87±0.56	2.09±0.46
	*V*_2up__Deg_velo						2.60±0.53	2.79±0.48	2.94±0.63	2.86±0.91	2.27±0.71	2.62±0.35	2.46±0.58	2.72±0.78
	Ext_Deg_Velo						2.29±0.35	2.36±0.43	2.48±0.52	2.44±0.55	2.09±0.53	2.20±0.28	2.19±0.47	2.37±0.41

*NF*: Non-fatigue condition; *F*: Fatigue condition; *H*: High lower limb mass ratio group; *L*: Low lower limb mass ratio group; Acc: Acceleration; Dec: Deceleration; ROM:Range of motion; Flex: Flexion phase; Ext: Extension phase; Deg_Velo: Degree (angular) velocity

*: Intervention group significant differences *p*≤0.05 (high *vs*. low); **Bold** rows represent; no significant (0.055 ≤ *p* ≤ 0.070) but had large effect size ($\eta_{\rho }^{2}$ > .14) interactions between fatigue condition.

There were significant differences between intervention groups (low vs high) at mean angle of hip (at *V*_downpeak_ and *V*_uppeak_); F_(1,19)_ = 7.29, p = .014, $\eta_{\rho }^{2}$ = .27, CI_%90_[0.03–0.486] and F_(1,19)_ = 13.88, p = .001, $\eta_{\rho }^{2}$ = .42, CI_%90_[0.129–0.599], respectively. The low group was significantly higher mean angles at *V*_downpeak_ and *V*_uppeak_ compared to the high group (mean diff. = 12.00° and mean diff. = 11.69°, respectively). There were also significant differences between intervention groups (low vs high) at shoulder and hip descent accelerations; F_(1,19)_ = 5.39, p = .008, $\eta_{\rho }^{2}$ = .15, CI_%90_[0.01–0.438] and F_(1,19)_ = 3.38, p = .1, $\eta_{\rho }^{2}$ = .14, CI_%90_[0.01–0.372], respectively. There were no other significant differences between the intervention groups (low vs high) depends on the region measurements (Table 1).

The main effect of all measurements’ mean and standard deviation are shown in Table 2. The statistically significant main effects (n = 21, non-fatigue vs fatigue condition) of linear and angular kinematic ANOVA results (above the box-whisker plots) are also shown in Figs 2–5.

**Table 2:** Results of within-subject effects by two-way mixed ANOVA.

Region			Joint					
			Shoulder (n = 21)		Hip (n = 21)		Knee (n = 21)	
			NF	F	NF	F	NF	F
Linear	*V*_1down_	m.s^-1^	0.67±0.18	0.76±0.25*	0.51±0.12	0.57±0.17*	0.18±0.08	0.21±0.10
	*V*_downpeak_		1.24±0.33	1.28±0.35	0.97±0.26	1.00±0.24	0.24±0.11	0.27±0.12
	*V*_2down_		0.80±0.19	0.85±0.23	0.62±0.17	0.67±0.18	0.13±0.11	0.12±0.07
	*V*_flex_		0.74±0.18	0.82±0.23*	0.56±0.15	0.62±0.16*	0.14±0.06	0.16±0.07*
	*V*_1up_		0.84±0.17	0.91±0.21^**ǂ**^	0.68±0.17	0.77±0.20*	0.13±0.05	0.15±0.08
	*V*_uppeak_		1.26±0.31	1.40±0.36*	1.05±0.28	1.19±0.27*	0.26±0.10	0.29±0.14
	*V*_2up_		0.70±0.21	0.82±0.27*	0.54±0.17	0.62±0.16 ^ǂ^	0.18±0.09	0.22±0.10*
	*V*_ext_		0.75±0.18	0.86±0.22	0.59±0.17	0.66±0.16 ^ǂ^	0.14±0.06	0.18±0.08*
	Descent_Acc	m.s^-2^	3.15±1.42	3.42±1.91	2.56±1.09	2.76±1.25	0.62±0.39	0.80±0.43
	Descent_Dec		3.53±1.42	3.68±1.54	2.74±1.14	2.91±1.26	0.62±0.34	0.79±0.35
	Ascent_Acc		3.82±1.61	5.14±1.95*	3.07±1.45	4.06±1.49*	0.97±0.73	1.00±0.44
	Ascent_Dec		3.15±1.22	3.82±2.12	2.68±0.83	3.09±1.51	0.62±0.31	0.77±0.54
Angular	*V*_downpeak__Angle	(°)	Not measured.		105.67±11.07	99.21±15.74 ^ǂ^	109.46±15.26	107.32±18.93
	*V*_uppeak__Angle				104.64±10.29	95.52±11.48*	109.77±16.09	103.86±16.21*
	ROM_Flex				100.93±12.97	103.77±14.41	90.60±16.91	96.42±18.57*
	ROM_Ext				100.47±13.06	101.32±13.51	90.95±16.22	95.13±17.70 ^ǂ^
	*V*_1down__Deg_Velo	rad.s^-1^ (ω)			2.71±0.65	2.9±0.86	2.41±0.42	2.60±0.56 ^ǂ^
	*V*_2down__Deg_Velo				1.78±0.42	1.64±0.66 ^ǂ^	1.56±0.40	1.71±0.09
	Flex_Deg_Velo				2.52±0.48	2.36±0.57	1.99±0.39	2.18±0.46 ^ǂ^
	*V*_1up__Deg_Velo				1.88±0.38	1.93±0.45	1.75±0.34	1.97±0.51 ^ǂ^
	*V*_2up__Deg_velo				2.69±0.51	2.9±0.76	2.43±0.58	2.58±0.68
	Ext_Deg_Velo				1.99±0.38	2.46±0.52	2.14±0.42	2.28±0.44

* *p* ≤ .05; ^**ǂ**^ *p* > .05, $\eta_{\rho }^{2}$ >.14; *NF*: Non-fatigue condition; *F*: Fatigue condition; Acc: Acceleration; Dec: Deceleration; ROM: Range of motion; Flex: Flexion phase; Ext: Extension phase; Deg_Velo: Degree (angular) velocity.

The test-retest reliability for linear kinematics of shoulder, hip and knee joints between the non-fatigue and fatigue conditions found that there was poor to good reliability (0.26 < ICC < 0.80) and there was poor to good reliability (0.08 < ICC < 0.79) for the angular kinematics of hip and knee joints between the fatigue conditions (Table 3).

**Table 3:** The test-retest reliability between conditions.

Region		Joint					
		Shoulder		Hip		Knee	
		Non-Fatigue—Fatigue Condition		Non-Fatigue—Fatigue Condition		Non-Fatigue—Fatigue Condition	
		ICC (*p*)	ICC 95% CI Lower- Upper	ICC (*p*)	ICC 95% CI Lower- Upper	ICC (*p*)	ICC 95% CI Lower- Upper
Linear	*V*_downpeak_	0.67 (0.001)	0.35–0.85	0.75 (0.001)	0.48–0.89	0.49 (0.01)	0.08–0.75
	*V*_flex_	0.73 (0.001)	0.45–0.88	0.80 (0.001)	0.58–0.91	0.74 (0.001)	0.46–0.88
	*V*_upeak_	0.60 (0.001)	0.24–0.81	0.59 (0.02)	0.22–0.81	0.62 (0.001)	0.27–0.83
	*V*_ext_	0.26 (0.12)^ǂ^	-0.18–0.61	0.39 (0.03)	-0.42–0.70	0.57 (0.003)	0.19–0.80
	Flex _Acc	0.69 (0.001)	0.38–086	0.71 (0.001)	0.41–0.87	0.38 (0.06)^ǂ^	-0.10–0.66
	Ext _Acc	0.33 (0.06)^ǂ^	-0.10–0.66	0.29 (0.09)^ǂ^	-0.15–0.63	0.04 (0.42)^ǂ^	-0.38–0.46
Angular	*V*_downpeak__Angle	Not measured.		0.47 (0.01)	0.59–0.74	0.75 (0.001)	0.49–0.89
	*V*_uppeak__Angle			0.43 (0.02)	0.007–0.72	0.64 (0.001)	0.30–0.83
	ROM_Flex			0.68 (0.001)	0.37–0.86	0.79 (0.001)	0.56–0.91
	ROM_Ext			0.79 (0.001)	0.55–0.91	0.66 (0.001)	0.34–0.85
	Flex_Deg_Velo			0.71 (0.001)	0.41–0.87	0.61 (0.001)	0.25–0.82
	Ext_Deg_Velo			0.38 (0.04)	-0.50–0.69	0.08 (0.35)^ǂ^	-0.35–0.49

^**ǂ**^:*p* > .05; other variables found statistically significant *p* ≤ .05; Acc: Acceleration; Dec: Deceleration; ROM: Range of motion; Flex: Flexion phase; Ext: Extension phase; Deg_Velo: Degree (angular) velocity

The main effect of exercise duration did not show statistically significant difference in the four regions (and in the flexion and extension phases) of the exercise at the different fatigue condition (*p* > .05) (Table 4).

**Table 4:** The main effect of the exercise durations for each region.

Time (s)	Region	Non-Fatigue	Fatigue
	*V*_1down_	0.43±0.15	0.42±0.15
	*V*_2down_	0.37±0.09	0.37±0.11
	*V*_flex_	0.80±0.18	0.79±0.15
	*V*_1up_	0.35±0.08	0.31±0.08
	*V*_2up_	0.41±0.08	0.44±0.14
	*V*_ext_	0.77±0.09	0.75±0.16
	Total-time	1.57±0.24	1.54±0.29

There was not found any significant main effect for the exercise durations, *p* > .05.

**Fig 6:**
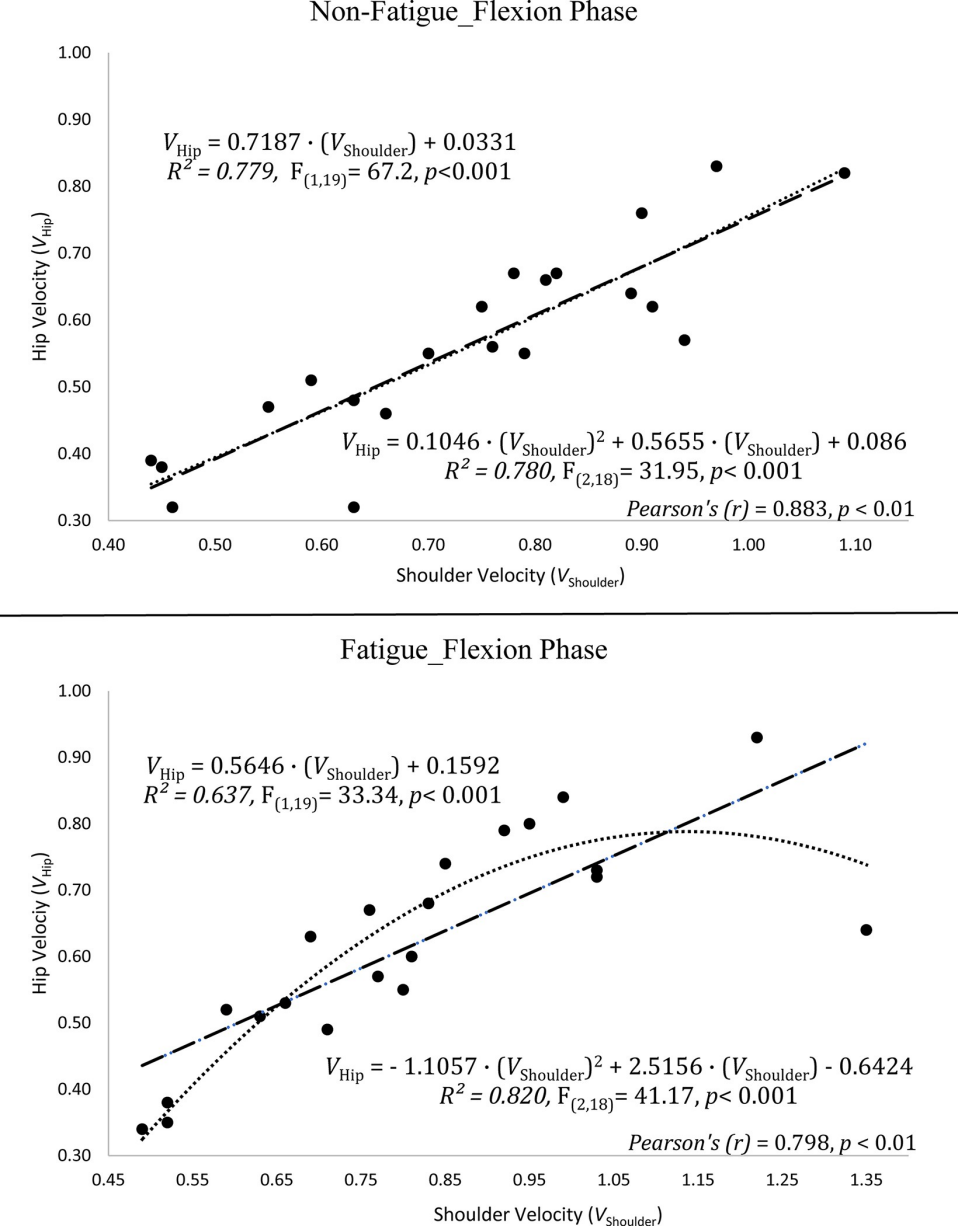
The relationship between the hip and shoulder joints in the different fatigue condition of the flexion phase.

**Fig 7:**
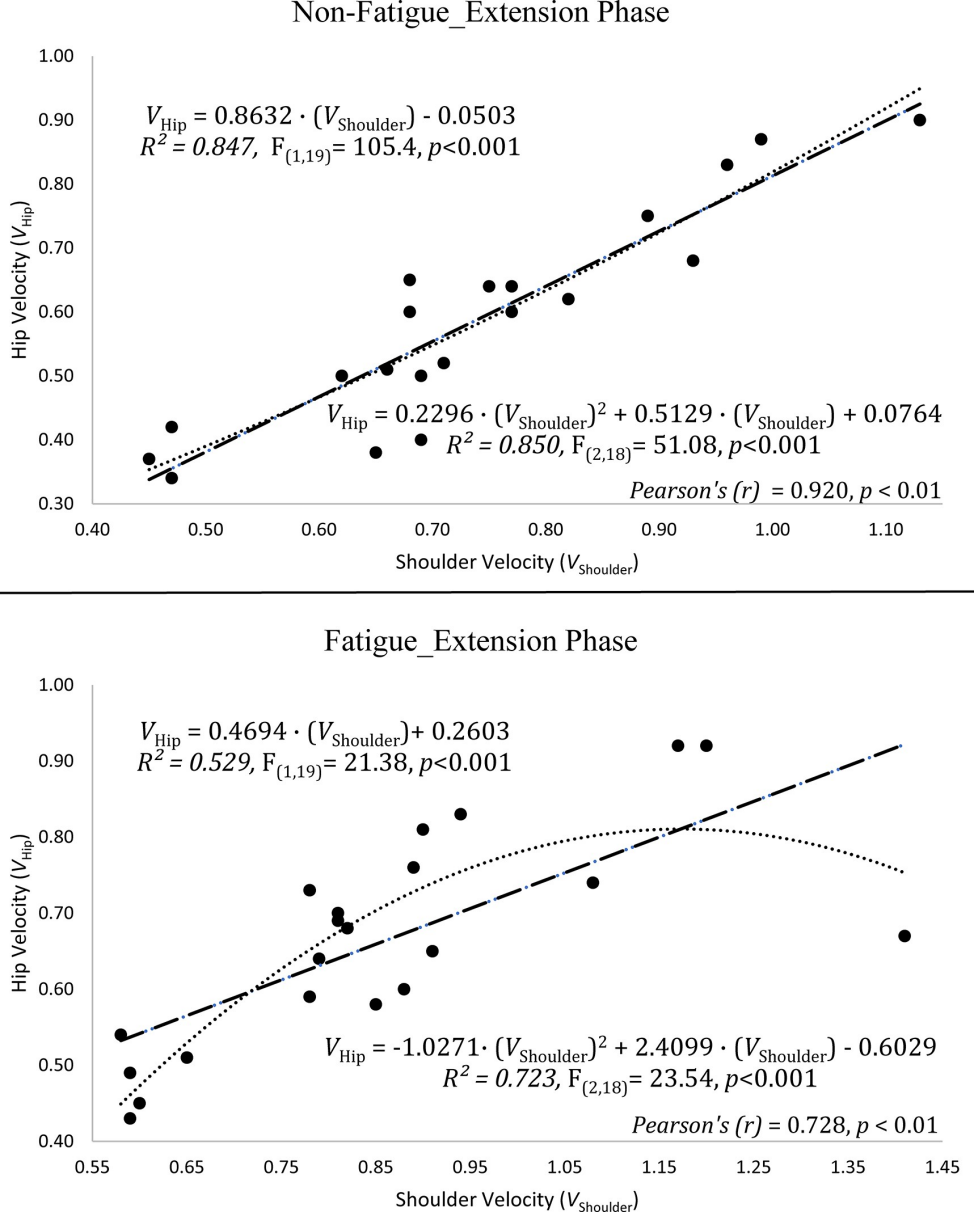
The relationship between the hip and shoulder joints in the different fatigue condition of the extension phase.

In the flexion phase, regression analysis of between velocity of hip and shoulder joints in the non-fatigue condition found that; R^2^_flex(linear)_ = 0.779, polynomial R^2^_flex(polynomial)_ = 0.78, Pearson’s (r) = 0.883 (p ≤ .01). and in the fatigue condition, R^2^_flex(linear)_ = 0.637, polynomial R^2^_flex(polynomial)_ = 0.82, Pearson’s (r) = 0.798 (p ≤ .01) (Fig 6). In the extension phase, regression analysis of between velocity of hip and shoulder joints in the non-fatigue condition found that; R^2^_ext(linear)_ = 0.847, R^2^_flex(polynomial)_ = 0.85. Pearson’s (r) = 0.92 (p ≤ .01) and in the fatigue condition, R^2^_ext(linear)_ = 0.529, R^2^_ext(polynomial)_ = 0.723. Pearson’s (r) = 0.728 (p ≤ .01) (Fig 7).

**Fig 8:**
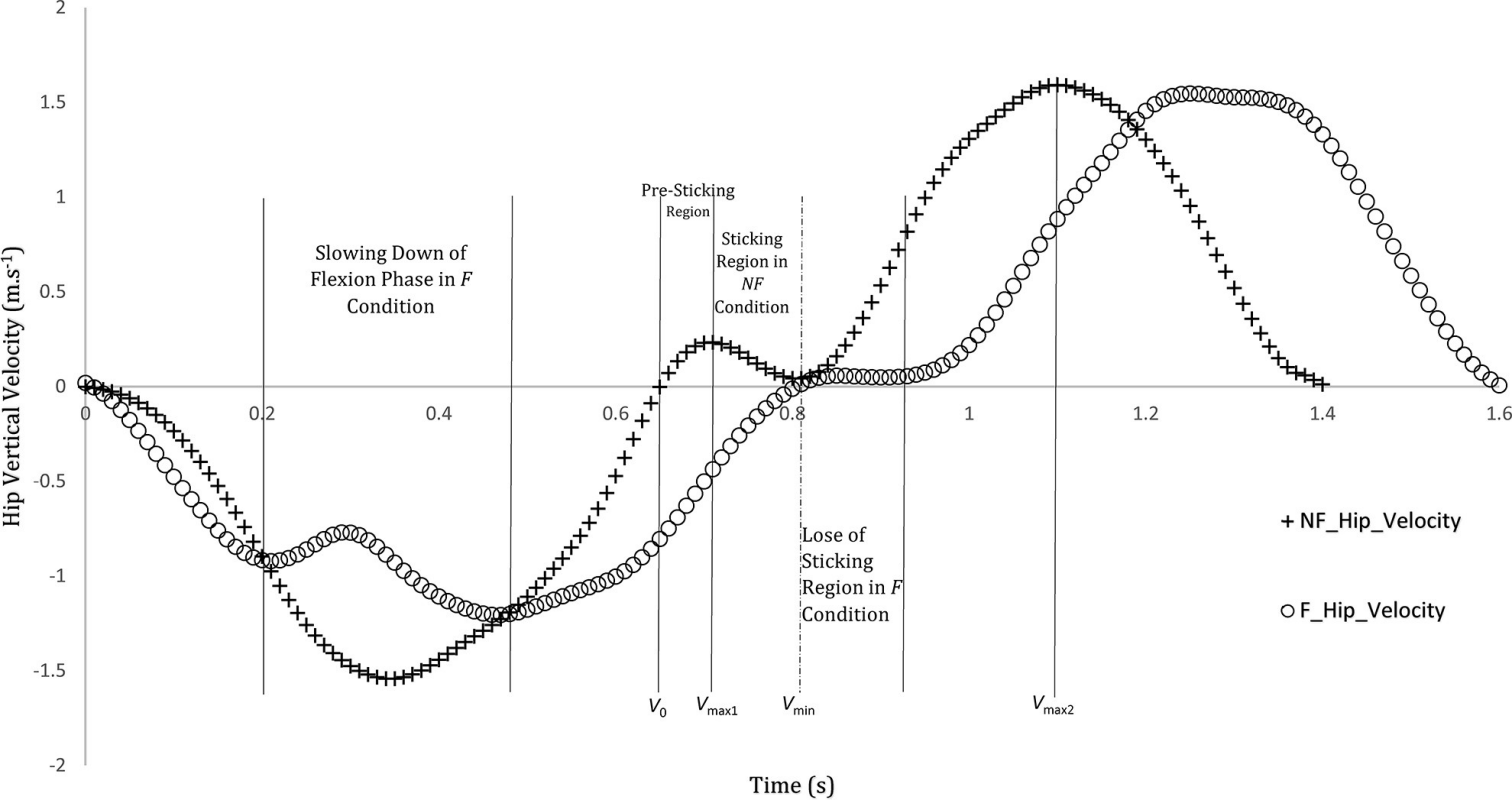
The vertical hip velocity in squats with an acceleration descent, deceleration descent, pre-sticking (between V0 and Vmax1), sticking (between Vmax1 and Vmin), and post-sticking region (between Vmin and Vmax2). F: fatigue condition, NF: non-fatigue condition.

**Fig 9:**
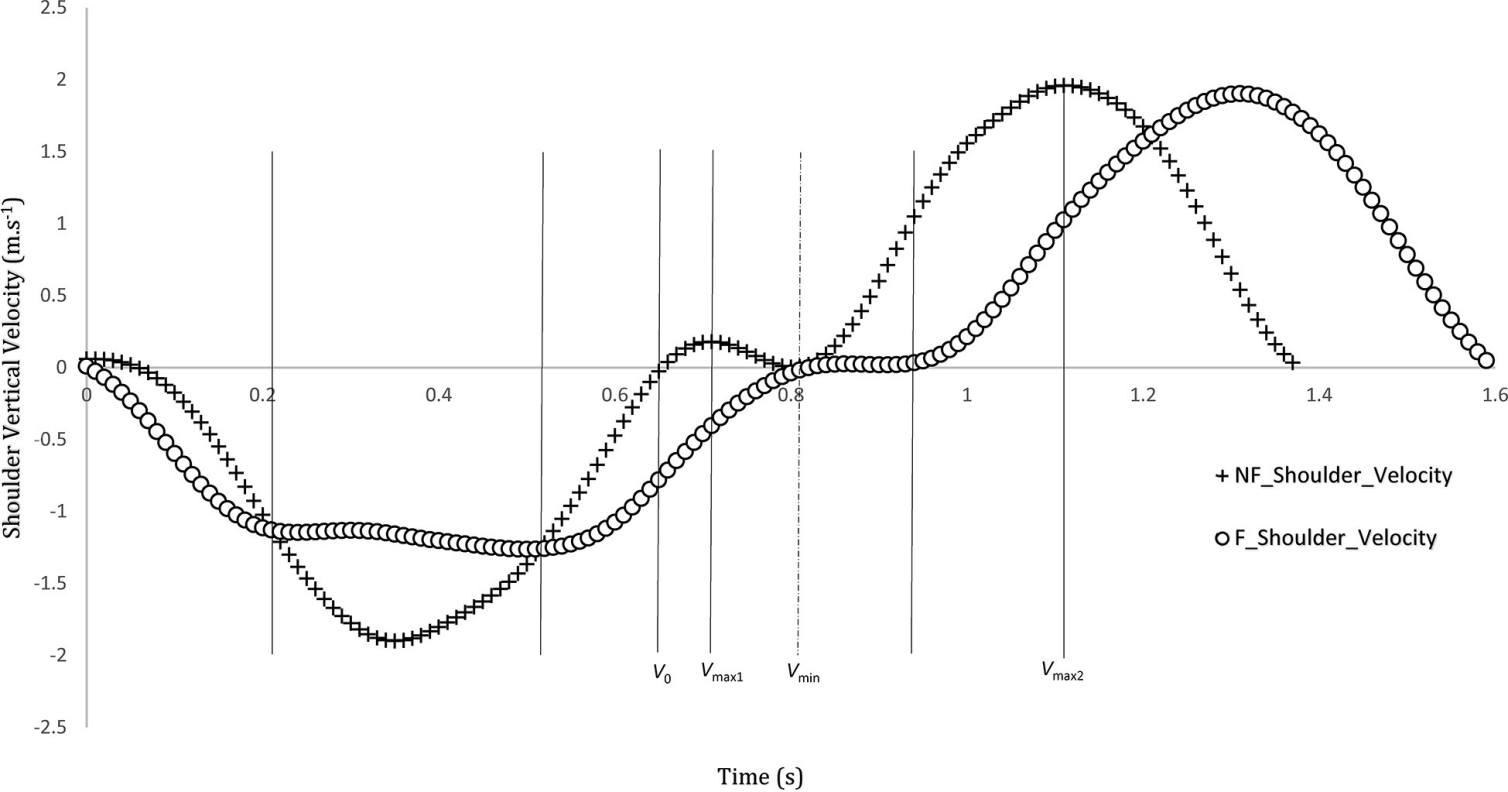
The vertical shoulder velocity in squats with an acceleration descent, deceleration descent, pre-sticking (between *V*_0_ and *V*_max1_), sticking (between *V*_max1_ and *V*_*min*_), and post-sticking region (between *V*_min_ and *V*_*max2*_*)*. F: fatigue condition, NF: non-fatigue condition.

The linear velocity of the shoulder and hip joints was drawn and examined for each participant. Meanwhile, it was found that one of the participants draw a curve similar to “sticking region”, and even in the non-fatigue condition (Fig 8), when the video analysis of this participant is examined carefully; it took exactly 0.06 s in the pre-sticking point (from *V*_0_ to *V*_max1_). Then, the time between *V*_max1_ and *V*_min_ were observed as 0.1 s in the sticking region. The ROM of the participant at the end of the flexion phase (*V*_0_); hip: 72°, knee: 67°, ankle: 67°, and the participant reached from *V*_max1_ to *V*_*min*_; hip: 72°, knee: 70°, ankle: 70° (Fig 8). There were also found similar curves for the shoulder joint as in shown Fig 9.

## Discussion

In this study, we investigated linear and angular kinematics of body weight squat exercise in two different groups according to their lower limb mass ratio (low and high) between the non-fatigue and fatigue conditions. They performed the exercise until exhaustion occurred. We showed that different lower limb mass ratio effects on kinematics of exercise. There was not observed any statistically significant duration difference at the different regions of exercise while there was significant different in the kinematics. In the fatigue condition, we showed that there was polynomial relationship between shoulder and hip joints. We posit that this could provide a new approach to velocity-based training. Additionally, our observations suggested that an individual’s habitual movement form during the squat exercise might contribute to the occurrence of sticking regions.

The mean flexion velocity was greater in the low group than the high group. It may be considered that the low group is trying to compensate the fatigue to comply with the tempo by increasing ROM during the extension phase. On the other hand, although the same trend was observed for the hip, no significant interaction was found, because the increases were less (ROM_Ext). Both groups are also tried to compensate for increased the initial phase of angular velocity (*V*_1down__Deg_Velo). This suggests that individuals with high lower limb mass ratio (LLMR) are more likely to be able to control more in the flexion phase during the body weight squat exercise (BSQ). It may be concluded that more caution should be taken with individual’s LLMR below 30% for BSQ.

### The main effects of angular and linear kinematics (n = 21, non-fatigue vs. fatigue condition)

Fatigue resulted in a decrease in the angle of peak velocity (*V*_downpeak__Angle) for both the hip and knee joints during the flexion phase. This indicates that in the fatigue condition, the hip reached peak velocity closer to the ground in this phase. This decreasing in the hip and knee is due to the fact that the participants performed the flexion phase (*V*_1down__Deg_Velo) with faster angular velocity (ω). In the flexion phase of fatigue condition, there was increasing in the angular velocities of the hip and knee joints in the initial acceleration region (*V*_1down__Deg_Velo) as well. However, during the deceleration phase (*V*_2down__Deg_Velo), the angular velocity of the hip decreased while the knee joint’s angular velocity increased. This may be due to the different mobility characteristic of the hip and knee joints.

In the non-fatigue condition, participants were able to perform a rapid flexion followed by a slower extension phase to keep up with the exercise pace. However, it resulted in an increase of 4.62% (flexion) and 23.58% (extension) in the fatigue condition. On the other hand, it is observed that mean flexion velocities (*V*_flex_) were less than the extension velocities (*V*_ext_). This condition particularly supports the effectiveness of fatigue during the flexion phase. Although the contraction phase (extension) is a general concern, especially in the studies related to velocity, this situation which increases even with the body weight, supports that should be more cautious in the flexion phase (descent) with heavy loads. In these conditions, it is usual to observe the increasing the load on the joints [3, 5, 33]. This situation may cause undesirable results in long term (i.e., injury). In the fatigue condition of the extension phase, decreased differences in the angles of peak velocity (*V*_uppeak__Angle) was observed in the hip and knee joints. This occurred when the angular velocity (*V*_1up__Deg_Velo) of the hip and knee joints increased in the extension phase. This increased velocity caused the peak velocity to be reached earlier in the extension phase. However, when the exercise durations were examined, neither acceleration (*V*_1down(flex.)_-*V*_1up(ext.)_; NF vs. F) nor braking (*V*_2down(flex.)-_*V*_2up(ext.)_; NF vs. F) phase did not show difference. In some studies, in order to avoid the effect of the stretch and shortening cycle at the end of the flexion phase (between eccentric and concentric phases), a short pause period is implemented approximately between 2–4 s [15, 34]. No such method was followed in the present study. Therefore, the observed increases in angular velocity may be attributed to the stretch-shortening cycle [35, 36] or the participants’ efforts to synchronize with the exercise pace. Another factor to consider is inertia. The contribution of the velocity in the extension phase to the increased in moment may be explained by faster flexion phase occurred (e.g., rebound effect of the movement). Hence, introducing a pausing period at the conclusion of the flexion phase could potentially yield positive effects, akin to isoinertial strength training, known for its performance-enhancing benefits [10, 37, 38]. The present study has been one of the few studies that observed to higher vertical linear and angular velocities in the fatigue condition. There were also increased difference in the flexion ROM of the hip and knee joints in the fatigue condition. This may be considered that participant try to keep themselves upright during the descent phase and release themselves faster due to the effects of gravity and fatigue. Another situation to consider was that in case of fatigue, the ROM of the knee joint would decrease during the descending phase, and the trunk could be excessive forward lean [39]. However, this possibility disappeared with the statistically significant increase in the flexion ROM of the knee joint without this increase in the hip joint.

In the flexion phase of the linear velocity, significant difference was observed in the shoulder, hip and knee joints. While this difference appears in linear velocities, no such difference was found in angular velocities (Flex_Deg_Velo) between fatigue conditions. However, flexion phase of the ROM supports this situation. While the linear velocity of the hip and knee showed difference in the extension phase, this was not observed in the shoulder joint. This suggests that participants exerted effort to maintain an upright posture during the fatigue condition. Otherwise, significant difference in this linear velocity could be observed during further forward and backward movements (i.e., excessive forward lean). Taken together, the non-significant difference in the ROM of hip supports this situation.

### The velocity relationship between shoulder and hip joints in the fatigue condition

In the studies of squat exercise related to velocity, it is generally performed by following the bar velocity [12, 14, 40–42]. The excessive forward lean of the trunk could be observed in the free weight back squat exercise [43], as a result of this, it might have a negative effect on the observation of the actual desired kinematics. Even in the non-fatigue condition, there was polynomial relationship between shoulder and hip with only body weight in this study. In addition, the linear relationship decreases more than the polynomial relationship in the fatigue condition. At the same time, the correlation coefficient between the velocities of the two joints in both conditions also decreased. In other words, fatigue condition in the extension phase caused further deterioration of the velocity relationship between the two joints. In fact, a study showed that monitoring bar speed for 1-RM with light loads did not show very strong linear equations as well [44], and Izquierdo et al. [45] found that parallel squat exercise had a significantly higher mean velocity in the last repetitions (60–75% 1-RM) than bench press exercise. In the present study performed with only body weight, hip velocities were lower than shoulder velocities in the each of the flexion and extension phases. According to present study, there may be more logical way to monitor hip joint instead of shoulder (bar) for velocity-based trainings and especially predict 1-RM with low-loads when fatigue occurred. Therefore, velocity-ranges [46] might be revised again for squat exercise or the desired exercise intensity would be observed more accurately. The present study raised doubts about observing the upper extremity for the squatting exercise may be considered as a partially indirect method. Therefore, the differentiation of the monitored region (bar vs hip) may allow better comparison of these velocities. This may give a new perspective for further studies. The lack of strong R^2^ (i.e., R^2^ < .95) prevented that generating a generally acceptable and more predictable power of the equations in this study. However, in the cases where the number of sample size is higher, it seems likely to find stronger predictable equations with higher R^2^ [40]. Although it is more logical and reliable to monitor bar speed for 1-RM estimation methods with heavy loads [44, 47–49], this may not benefit the same rate in velocity-based squat trainings in general. The differences in these velocity outputs (hip and shoulder) can be considered quite normal that the joints take different distance at the same period of time. However, the situation that needs to be emphasized here was the R^2^ and coefficient correlation (r) values which are changed in the different fatigue conditions. This situation, together with the external load, may cause less difference in the shoulder and hip joints. However, in an exercise with free weight, excessive movement of the trunk in the fatigue condition may prevent us from observing the desired velocity from shoulder (bar). According to this study, it is more appropriate and predictable for the relationship between shoulder and hip joints had non-linear relationship with a polynomial curve after fatigue occurred.

### Is it possible to observe “sticking region” for BSQ?

The “sticking region” could be defined as deceleration and re-acceleration when lifting the bar with additional load in the contraction phase (moving upwards) during exercise (i.e., back squat, bench press) and usually observe before completing the movement, which may be result of exhaustion/inability to perform the exercise [13, 40, 50, 51]. In this study, it is observed that one of the participants draw a curve similar to “sticking region” with only body weight and this happened within just 3° and 0.16 s (from *V*_0_ to *V*_*min*_*)*. Therefore, it was not noticed by the researchers during the test that the participant had such a tendency. It is surprising that was observed without fatigue in the BSQ, although sticking region in the literature is generally occurred at the end of exercise (last repetition) with additional loads, and in some cases not even observed [13, 14].

Another interesting aspect is that during the flexion phase in the fatigue condition, a similar slowdown is observed while descending. This may be a situation-specific to the hip joint and this is observed in the graphics of some studies. Kristiansen et al. [52] showed that there is no generally acceptable optimal lifting method, each individual may find their own way to successful lifting and individual differences may affect performance positively such as shorter trunk and thigh length. In this study, the participant whose curve is drawn, did not have any disability for proper form of the BSQ. It has been observed that both joint (shoulder and hip) draw similar curves. It may confirm the speculation about the monitoring of velocity in this study. Although they had similar tendencies, as a result of a detailed examination, a linear relationship was not observed between two joints. Also, observing the sticking region in the non-fatigue condition of BSQ, it may arise the possibility that this phenomenon may occur participant’s form of squat (unconsciously happening, performing exercise in their own way). This situation, which corresponds to 4.76% of the participants in the present study, considering that it is performed with external load, may increase the possibility of observing the sticking region. R. van den Tillaar et al. [53] found that this region observed in two-thirds of the participants in 6-RM squat exercise. Martínez-Cava et al. [54] found that sticking region was not occurred in the half squat even with heavy loads. Perhaps before testing this phenomenon with external loads, the tendency of participants to such situation may be compared by performing a pre-test examination with the BSQ.

### Practical applications

The lower limb muscle ratio differences may lead to significant interactions for angular kinematics in the fatigue condition. If individuals have less than 30% LLMR may increase the risk of movement controlling especially in the flexion phase after fatigue occurred. In this sense, it may be important to calculate the LLMR before squat exercises. The relationship between the shoulder and hip joints may be better explained by polynomial rather than linear relationship in the fatigue condition. Because of this situation, tracking the hip joint may provide an important recommendation for observing squat exercise in the velocity-based training. The studies with higher number of participants may contribute more for the future researches about this topic. Depends on the present study, it may be necessary to pre-test with BSQ before studies for observing the sticking region. Therefore, individuals who tend to show sticking region may be identified and the studies carry out with this method may provide more contribution as to why this phenomenon occurs.

## List of abbreviations

BSQ: Body Weight Squat
LLMR: Lower Limb Muscle Ratio
ROM: Range of Motion
*V*_ext_: Extension Phase of Joint Velocity
ROM_ext_: Extension Phase of ROM
*V*_flex_: Flexion Phase of Joint Velocity
ROM_flex_: Flexion Phase of ROM
*V*_1down_: Region of beginning of flexion (full extension position of knee, First Region)
*V*_downpeak_: Peak velocity of flexion phase
*V*_2down_: From peak velocity of flexion (*V*_downpeak_) to the end of flexion (Second Region)
*V*_1up_: The third region started from beginning of extension phase to peak extension velocity (*V*_uppeak_)
*V*_uppeak_: Peak Velocity of Extension Phase
*V*_2up_: Forth region was from peak velocity of extension (*V*_uppeak_) to end of extension phase
